# Nature-based mind–body intervention for test anxiety in adolescents: a feasibility study

**DOI:** 10.3389/fpsyg.2025.1550353

**Published:** 2025-04-09

**Authors:** Xuan Zeng, Yarui Zhang, Ziyan Chu, Tianyong Chen

**Affiliations:** ^1^State Key Laboratory of Cognitive Science and Mental Health, Institute of Psychology, Chinese Academy of Sciences, Beijing, China; ^2^Department of Psychology, University of Chinese Academy of Sciences, Beijing, China; ^3^Institution for Brain and Psychological Science, Sichuan Normal University, Chengdu, China; ^4^College of Liberal Arts & Sciences, University of Illinois Urbana-Champaign, Urbana, IL, United States

**Keywords:** nature-based mind–body intervention, test anxiety, adolescent, anxiety, depression

## Abstract

**Introduction:**

Traditional interventions for test anxiety often fall short of addressing the specific needs of adolescents and educational institutions due to issues of stigmatization and professionalization. In contrast, nature-based interventions (NBIs) have gained traction for their potential to enhance mental health, as they are generally accepted and cost-effective, thereby offering a promising alternative for alleviating test anxiety.

**Methods:**

This study seeks to investigate the feasibility of a nature-based mind– body intervention (NMI) designed to reduce test anxiety among adolescents by integrating elements of NBIs and mind–body therapies (MBTs).

**Results:**

The findings suggest that NMI can significantly diminish excessive test anxiety and academic stress, along with alleviating symptoms of general anxiety and depression.

**Discussion:**

This approach presents a low-stigma, low-cost strategy for managing test anxiety in adolescents and offers valuable insights for mental health policymakers. Furthermore, it may enhance academic performance and broaden educational access for disadvantaged populations in developing countries.

## 1 Introduction

“Test anxiety is anxiety related to situations where abilities or knowledge are being tested (Medical Subject Headings, 2021).” It is a universal phenomenon among adolescents, and people from diverse cultural backgrounds worldwide have similar experiences (Zeidner, 2020). Although tests can enhance learning and metacognitive insights (Yang et al., 2021), excessive test anxiety caused by high-stakes tests can obstruct academic performance (Lowe et al., 2008). Excessive test anxiety could impact an individual’s behavior, cognition, and physiological functioning. It may trigger task-avoidance actions, manifest worry, cognitive interference, and fear of social humiliation, and often lead to a state of over-arousal, such as sweaty palms, increased heart rate, and shallow or rapid breathing in examination situations (Lowe et al., 2008). At the neural level, test anxiety could increase the effects of stimulus-driven attention systems and impair the function of goal-directed attention systems (Hu et al., 2023). Test anxiety is highly associated with more severe mental illnesses such as anxiety and depression (Robson et al., 2023; Wuthrich et al., 2020), reciprocal relations with elevated risk for developing emotional disorders (Putwain et al., 2021). In Western society, it is estimated that close to 25–40% of adolescents suffer from test anxiety (Zeidner, 2020), and the prevalence in China is up to 46.7–72.43% (Chen et al., 2023; Jian et al., 2022; Shen et al., 2018). The high prevalence rate reflects a highly competitive academic environment, including pressure from a test-based education system and related to parental expectations, which is one of the reasons often cited to explain mental health problems among adolescents in China (Qiao et al., 2021).

Conventional interventions for adolescent test anxiety primarily include behavioral therapy, cognitive-behavioral therapy (CBT), and pharmacological treatment (Huntley et al., 2019; Zeidner, 2020; Putwain and von der Embse, 2021; Szeleszczuk and Frączkowski, 2022). Behavioral therapy employs systematic desensitization and relaxation training to help adolescents gradually adapt to test situations and alleviate anxiety symptoms (Aihie and Igbineweka, 2018; von der Embse et al., 2012). However, its effects may be limited to specific contexts and not sufficiently address cognitive aspects. CBT combines cognitive restructuring with behavioral training to modify irrational thoughts and enhance self-efficacy. Still, it often requires a longer treatment duration and may be less effective for severe anxiety cases (Putwain and von der Embse, 2021). Pharmacological treatments, such as anti-anxiety and antidepressant medications, provide rapid symptom relief but carry potential side effects and dependency risks and fail to address the underlying psychological causes of anxiety (Szeleszczuk and Frączkowski, 2022). Recent meta-analysis evidence discovered that mind–body interventions have a more positive effect compared to other intervention methods in alleviating adolescents’ test anxiety (Zeng et al., 2023). Mind–body therapies (MBTs) are treatment methods or techniques based on the knowledge of mind and body interactions (Medical Subject Headings, 2009), such as mindfulness and Tai Chi (Gouda et al., 2016; Guo and Sun, 2019; Li and Li, 2017). It can alleviate tension, mitigate the impact of stress, enhance emotional well-being, and promote overall health (American Academy of Pediatrics, 2016). There is another obvious advantage of MBTs, compared to psychotherapy and psychopharmacology, MBTs have less stigma, making them more suitable for adolescent groups in schools (Bandealy et al., 2021; Kohen, 2009). Nevertheless, there are thresholds for realizing these benefits and advantages because of the requirement of professional guidance. For example, a complete mindfulness intervention usually spans 8 weeks to 4 months and is led by a qualified counselor (American Academy of Pediatrics, 2016) with an additional burden for schools to use regularly. It is unknown whether MBTs can have similar effects on test anxiety if they are simplified to reduce reliance on professional guidance.

Recently, nature-based interventions (NBIs), a promising sustainable intervention, have garnered attention. NBIs are defined as programs, activities, or strategies designed to engage people in nature-based experiences with the explicit objective of improving health and well-being outcomes (Shanahan et al., 2019; Shrestha et al., 2025). Since 1979, Ulrich first confirmed that viewing natural landscape pictures can reduce college students’ exam stress under laboratory conditions (Ulrich, 1979), NBIs have gained widespread use in addressing stress (Johansson et al., 2022), physical health (Fan et al., 2023), and mental health (Cuthbert et al., 2021) by using the natural environment, nature-based activities, or promoting nature appreciation (Wang et al., 2024). According to the theory of Wilson (1986) proposed “Biophilia” - the human instinct to get close to the natural world as a necessary biological basis for individual development. Explained why NBIs are relatively easy to accept. The earlier Stress Reduction Theory (SRT; Ulrich, 1983) and Attention Restoration Theory (ART; Kaplan and Kaplan, 1989) explained how nature reduces somatic and psychological stress and relieves directed attention fatigue. Stress and attention are also the core issues of test anxiety intervention. Test anxiety is often accompanied by high levels of stress, manifested by physiological arousal (e.g., increased heart rate, sweating) and negative emotions (e.g., nervousness, fear). It also consumes many attention resources, resulting in distraction, memory loss, and impaired cognitive function. These theories provide the scientific basis for NBIs to design and implement effective interventions to promote mental health. NBIs have the characteristics of low cost and low stigma. As a cost-effective complementary medical treatment, it can reduce the overall medical costs of individuals and society (Harrison et al., 2023; Tambyah et al., 2022). Moreover, engagement in NBIs can help reduce the stigma surrounding mental illness and its treatment, fostering open communication among participants (Masterton et al., 2020; van den Berg and Beute, 2021). NBIs cover a variety of forms, such as nature walks (White et al., 2023), forest bathing (Kotera et al., 2022), community gardening (Litt et al., 2023), and MBTs in natural environments such as mindfulness (Djernis et al., 2023). However, the current application of NBIs is limited. For example, their implementation in mental health institutions remains sporadic (Tambyah et al., 2022), and they have not yet been extended to academic-related fields (see Supplementary material). As a result, both adolescents and institutions lack the opportunity to benefit from the advantages of NBIs.

In summary, traditional test anxiety interventions often fail to meet the needs of adolescents and educational institutions due to stigma and professionalization. In contrast, NBIs have significant potential for health promotion and sustainability, while the MBTs have the advantages of low stigma and acceptability. Given this, our study combined the two methods, the nature-based mind–body intervention (NMI), to explore the feasibility and acceptability of application in adolescent test anxiety. Furthermore, we also assessed alterations in general anxiety and depressive symptoms pre-and post-intervention to measure the influence on participants’ mental health.

## 2 Materials and methods

### 2.1 Design

Due to practical constraints and control group limitations (match failure), and informed by prior research, this study adopted a single-arm intervention design with convenience cluster sampling (Uzun et al., 2024; Aytaç and Bayram, 2024; Liu et al., 2024). The selected schools were chosen based on three factors: (1) schools with limited educational and health resources in underdeveloped regions, (2) the feasibility of implementing the intervention in the final year of middle school or the final year of high school, and (3) the similarity of the subjects’ demographic information. One boarding school for left-behind children met the criteria and agreed to conduct NMI in the third grade of junior high school (Grade 9).

The NMI study runs from October 2020 to May 2021 and includes six three-hour sessions. These sessions were conducted monthly or bimonthly. Three self-rating scales were collected 1 week before the initial phased examination and used again 2 weeks before the high school entrance examination. After the sessions, the participants underwent qualitative analysis through interviews. The session implementers were unaware of the research’s purpose, and the research designers did not participate in implementing the course.

### 2.2 Participants

The Participant’s school is a 12-year boarding school in the township, including primary, middle, and high school. All the students come from neighboring cities and towns, and 83.7% have parents who have gone out for work or are missing as shown in Table 1. In other words, most of them are left-behind children. They represent those disadvantaged groups in economically underdeveloped areas that lack adequate educational and mental health care resources. They will take the high school entrance examination at the end of the school year, which is both a measure of the academic level of middle school and an entrance selection test for high school. Usually, only 50% of students can pass the examination to continue general education, and the rest will enter vocational education or flow into society.

**Table 1:** Demographic description of the participants at baseline.

Items	Total (*n* = 129)	Groups (TAI)				
		Excessive (*n* = 40)	Moderate (*n* = 42)	Without (*n* = 47)	*χ^2^*	*p*
Age	14.53 ± 0.75	14.45 ± 0.68	14.62 ± 0.76	14.53 ± 0.80	0.52	0.598
Female (%)	31.00	30.00	33.30	29.80	0.16	0.924
Left-behind children (%)	83.7	90.00	85.71	76.60	3.03	0.220
Willingness to enter high school (participants) (%)	93.00	90.00	97.60	91.50	3.87	0.423
Willingness to enter high school (parents) (%)	96.90	97.50	97.60	95.70	1.77	0.778

TAI, Test Anxiety Inventory.

The study included 129 participants (89 boys and 40 girls) with a mean age of 14.53 ± 0.75 years. Participant attrition was attributed to relocation and school transfers. No students reported having any medical diagnoses of anxiety, depression, or learning disabilities. Following the administration of the Test Anxiety Inventory (TAI) during the baseline assessment, participants were categorized into three groups based on their test anxiety levels: excessive test anxiety, moderate test anxiety, and without test anxiety. Demographic details for each group are presented in Table 1.

### 2.3 Materials

#### 2.3.1 Test anxiety inventory

The American clinical psychologist Spielberger developed the TAI in 1980 (Spielberger and Gonzalez, 1980). The Chinese version of TAI was introduced in 1987. It is a self-report scale consisting of a total of 20 questions. The questions are divided into two-factor subscales: “Worry” and “Emotionality,” with eight questions each. Additionally, four questions do not belong to either factor. The Likert scoring method determines the degree of specific signs of anxiety an individual feels in an examination situation. The scoring ranges from 1 to 4, with 1 representing “never,” 2 representing “sometimes,” 3 representing “often,” and 4 representing “always.” Higher scores indicate higher levels of test anxiety.

Based on previous research (Broks et al., 2023; Putwain and Daly, 2014), the upper third of subjects’ scores were classified as excessive test anxiety, the middle third as moderate test anxiety, and the lower third as without test anxiety.

#### 2.3.2 The screen for child anxiety-related emotional disorders

The SCARED form is a screening tool designed for children and adolescents aged 9 to 18 years to self-assess anxiety disorders (Birmaher et al., 1997). The form was localized into Chinese in 2002 and a norm was established. The scale is made up of 41 items grouped into five factors: somatization/panic, generalized anxiety, separation anxiety, social phobia, and school phobia. The items are scored on a three-level scale from 0 to 2, with 0 indicating no such problem, 1 indicating sometimes present, and 2 indicating often present. A total score of 23 or higher may suggest an anxiety disorder.

#### 2.3.3 Depression self-rating scale for children

The DSRSC is a depression assessment tool developed to assess depression in children aged 8 to 16, based on the diagnostic criteria for adult depression (Birleson, 1981). In 2003, the Chinese version of the scale was revised, and a norm was established. The scale consists of 18 items, and each item is rated on three levels: not (0), sometimes (1), and often (2). A total score of 15 or higher may suggest a depressive disorder.

### 2.4 Process and procedures

The intervention was conducted in a naturalistic environment, the Ying River, 200 meters from the school. The site is an open area undergoing municipal environmental renovation, with limited natural vegetation despite some greenery along the riverbank. Pedestrians are scarce and vehicles are impassable. The river is vast, and the opposite bank features rural wilderness with green spaces. Ying River, which the locals revered as the mother river. Drawing inspiration from the seasoning river and the lush greenery lining its banks, we designed a tailored NMI called ‘River Song’. Every session is divided into introduction, expansion, and summary sharing. Table 2 presents the content of the six sessions and the NBIs and MBTs used. The NBIs included nature play, field observation, nature walks, and nature craft/art. Meanwhile, the MBTs included meditation, relaxation, expressive writing, psychodrama, and imagery. Two types of techniques have been validated through quantitative studies by other studies (Daniels et al., 2022; Sprague and Ekenga, 2022; Zeng et al., 2023; Kwon and Lee, 2024). The intervention is arranged in mental health classes and self-study classes, and the periods are continuous through class adjustments without affecting the teaching plans of other subjects.

**Table 2:** Session content and techniques.

Session	Theme	Objective	Content
First	“Who I am”	Learn the principle of natural activities; Understand oneself; Build mutual trust; Familiarize oneself with nature and build natural connections.	Nature play: Who I am^a1,b1^ Observe nature, name oneself with natural things, and act it out for others to guess. Vipassana: Fly in your sky^b2^ Learn to focus on one’s inner strength and feel one’s real existence. Field observation: River life^a2,b1^ Understand life through in-depth observation of rivers. Throw cobblestones^b3^ Tell the stone unhappiness, then hurl it into the river. Afternoon tea Summary: Write cards^b4^ Write down activity feelings and express feelings toward peers.
Second	“Follow along on the river journey”	Positive feedback boosts confidence.	Vipassana: Fly in your sky^b2^ Relax and feel the vitality of natural life. Throw cobblestone^b3^ Tell the stone unhappiness, then hurl it into the river. Psychodrama: The Story of the River^b1,b5^ Each person plays a natural role of river, connects roles into a storyline, and performs it theatrically. After the show, share the support provided for peers and appreciate the support gained. Afternoon tea Meditation^b2^: Look back on 2020, be thankful for what you have now, and empower yourself; Summary: Write thanks card^b4^ Write down activity feelings and give thanks (to yourself, peers, life.).
Third	“The Sunrise”	Appreciate each other as the sunrise. Learn to praise others.	Nature walks^a2,a3^: Hike 7 kilometers south along the Quan River from the school and see the sunrise. Throw cobblestone ^b3^ Tell the stone worry, then hurl it into the river. Collect praise cards ^b4^: Collect signatures from 10 peers who are willing to praise you.
Fourth	“Search for Spring”	Feel the spring; Learn to praise others and accept praise in return.	Natural play^a1,b4^: Find the natural scene based on the assembled puzzle, and express feelings during the process through origami poetry. Afternoon tea Vipassana: Fly in your sky^b2^ Feel inner emotions A Xiǎo Hóng Huā for you*,^3,b4^: Write down one’s strengths and send three Xiǎo Hóng Huā to peers.
Fifth	“Unique life”	Deepen nature connection; Like nature, anyone is unique, not alone, and can do more.	Nature Walks^a3^, Field observation^a2^: Through the Gate of Life (Landscape Transition Zone), experience the diversity of nature on both sides of the river in spring. Nature craft/art^a4^ Collect natural items to prepare for making crafts next time. Picnic Vipassana: Fly in your sky^b2^ Feel the flow of your inner emotions Summary: The class head teacher gave a card in advance written to each student and a hug. River Concert^b1,a4^: Improvise with the river as the theme.
Sixth	“My world, my choices”	Figure out what you truly desire to do; Self-motivation.	Nature Walks^a3^ Hike 2 kilometers south along Quan River from the school to the Large Lawn (session location). Painting^a1^: First, describe your appearance, then invite 10 peers to draw your portrait based on the description. Throw cobblestone^b3^ Tell the stone stress, then hurl it into the river. Vipassana: Fly in your sky^b2^ Flowing forward like a river. Summary^a4,b4^: Write down the most important things in 3 years next to the self-portrait and bless yourself; Draw your future with the craft of natural items collected in the previous unit.

Nature-based intervention: a1, nature play; a2, field observation; a3, nature walks; a4, nature craft/art. Mind–body therapies: b1, imagery; b2, meditation; b3, relaxation; b4, expressive writing; b5, psychodrama.

For example, in session 1, students play a nature game called “Who am I?” The game involves observing the natural environment and choosing a natural object as a name. For example, some students named themselves after the fast-growing poplar trees on the banks of the Quan River. They gave themselves a pun name like “Super Poplar” (in Chinese, Poplar and the surname Yang are the same Chinese character). By natural names, students reflect on their relationship with nature and create a new image of natural objects through their unique emotional activities. The display link conveys the emotion or information they gave the name to, such as the strength and exuberant vitality of “Super Poplar.” The principle of natural activities refers to goals aimed at achieving through natural activities, including respecting life, caring for nature, living simply, appreciating resources, fostering positive relationships, and prioritizing physical and mental well-being. In session 4, origami poetry is a collaborative activity where participants write one line of a poem on paper, fold it to conceal their line, and pass it on. Once everyone contributes, the paper is unfolded to reveal the complete poem, blending creativity, surprise, and teamwork into a shared artistic experience. “Xiǎo Hóng Huā,” a small red flower in Chinese culture, symbolizes praise, encouragement, and recognition. Often given to children for good behavior or achievements, its red color represents happiness and luck, while the flower signifies growth and potential, inspiring continued progress.

### 2.5 Measures and statistics

The test is conducted in class using paper and pencil and takes approximately 30 min. Two experimenters were responsible for administering the test. After the students completed their questionnaires, the experimenters reminded them to review their work for errors or omissions before collecting the forms. The questionnaires were entered and coded using Excel, and invalid questionnaires were eliminated. All data were analyzed statistically using SPSS 23. Pre-test and post-test comparisons were conducted on test anxiety, anxiety symptoms, and depressive symptoms. The standardized mean difference, Hedges’ *g*, a modification of Cohen’s d, is used to measure effect size. The effect size is expressed using *g* = 0.2, 0.5, and 0.8, to small, medium, and large effect sizes, respectively (Kallapiran et al., 2015).

### 2.6 Interview analysis

After the intervention, a qualitative analysis was conducted, and participants were surveyed on their attitudes, feelings, reactions, and more. Each participant was given ample time to complete a questionnaire individually. The qualitative data was analyzed using NVivo 12, and conclusions were drawn through bottom-up secondary coding. These conclusions were verified by repeatedly referring to the original materials.

## 3 Results

### 3.1 Analyze the scores of pre- and post-tests for various levels of test anxiety

**Figure 1:**
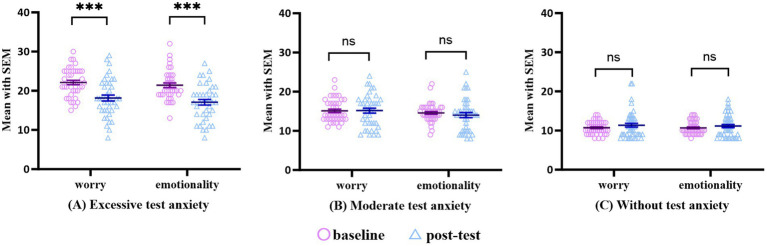
The comparison of test anxiety factors pre- and post intervention. ***, *p* < 0.001; ns, no significant difference.

A paired sample *t*-test was conducted on the pre-and post-test results of the excessive, moderate, and without anxiety symptom groups, along with the effect size calculation, to explore the changes in test anxiety at different levels before and after the intervention. Table 3 indicates that the results show a statistically significant difference between the excessive test anxiety group where the effect size was large. In contrast, the moderate test anxiety group and the without test anxiety group were not statistically significant after the intervention. Further observation of the changes in the two factors of test anxiety, Worry, and Emotionality, before and after the intervention (Figure 1) showed a trend consistent with the total score. The difference in Worry (*t* = 4.52, *p* < 0.001, *g* = 0.93) and Emotionality (*t* = 6.11, *p* < 0.001, *g* = 1.04) factors in the excessive test anxiety group were statistically significant. In contrast, the difference in the two factors between the moderate test anxiety group and the without test anxiety group was not statistically significant.

**Table 3:** Comparison of test anxiety group pre-and post-intervention.

Group	Baseline	Post-test	*t*	Hedges’ *g*
Excessive test anxiety (*n* = 40)	53.80 ± 7.80	44.13 ± 10.50	5.74***	1.05
Moderate test anxiety (*n* = 42)	37.55 ± 3.88	37.19 ± 9.27	0.28	0.05
Without test anxiety (*n* = 47)	28.11 ± 3.03	29.00 ± 6.53	−0.98	−0.18

Two-trail test; ^***^p < 0.001.

### 3.2 Pre- and post-test analysis of anxiety and depressive symptoms

**Figure 2:**
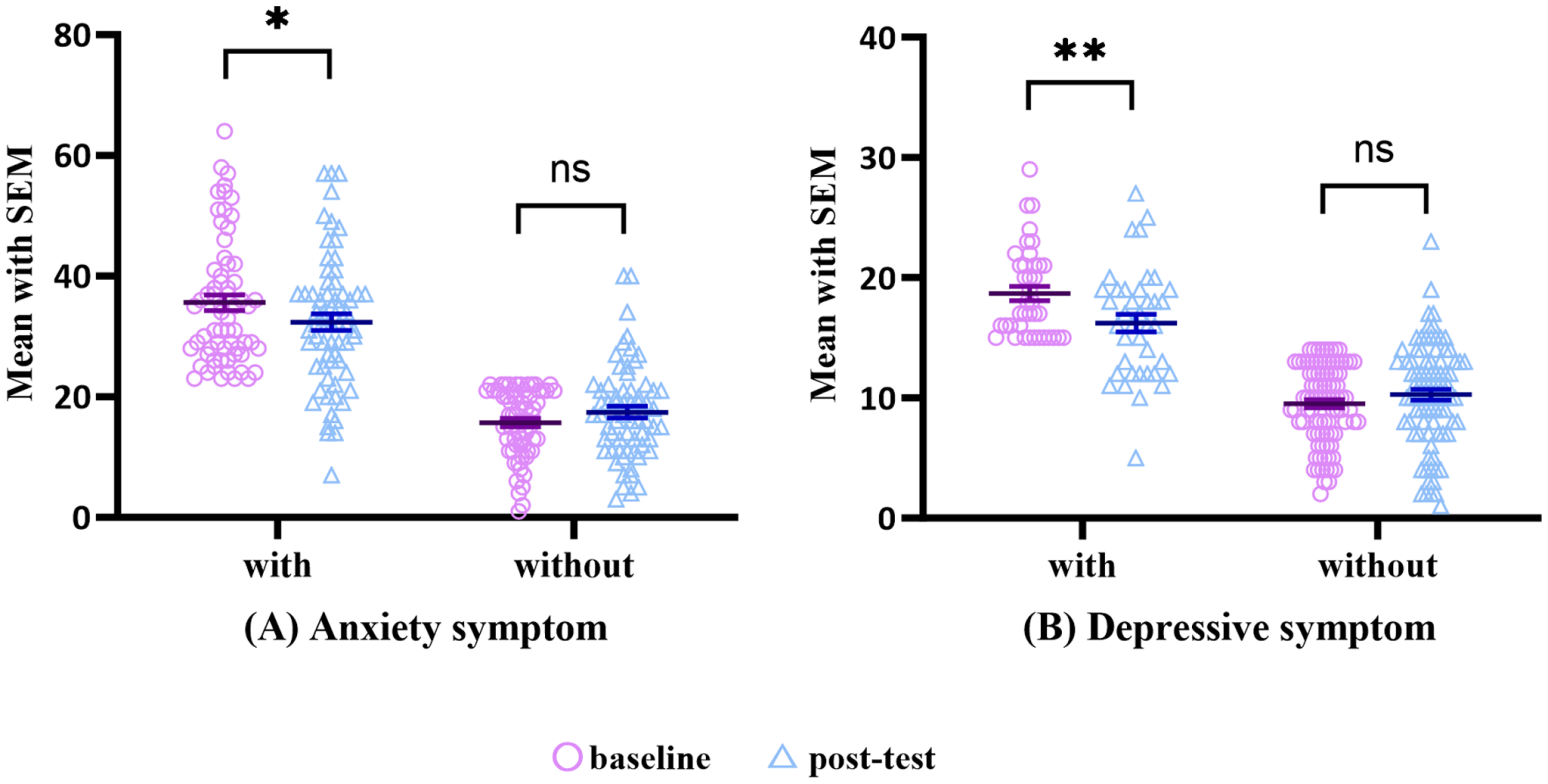
The comparison of anxiety and depressive symptoms pre- and post intervention. *, *p* < 0.05; **, *p* < 0.01; ns, no significant difference.

To analyze the impact of the intervention on anxiety and depressive symptoms, we recorded the proportion and scores of subjects experiencing these symptoms before and after the intervention. Our findings, presented in Table 4, showed that at the baseline stage, 50.38% of all subjects had anxiety symptoms, while 30.23% had depressive symptoms. Additionally, the severity of test anxiety was found to have a statistically significant effect on the proportion of subjects experiencing anxiety and depressive symptoms, as shown in Figure 2. After the intervention, those with anxiety symptoms (*n* = 65, *t* = 2.30, *p* = 0.025, *g* = 0.30) and those with depressive symptoms (*n* = 39, *t* = 3.55, *p* = 0.001, *g* = 0.59) both exhibited a significant decrease. The changing trend of general anxiety and depressive symptoms before and after the intervention closely resembled that of excessive test anxiety. However, patients without anxiety or depressive symptoms did not show significant differences in their total scores before and after the intervention.

**Table 4:** The proportion of symptoms of anxiety and depression at baseline.

Items	Total (*n* = 129)	Groups (Test anxiety)				
		Excessive (*n* = 40)	Moderate (*n* = 42)	Without (*n* = 47)	*χ^2^*	*p*
With anxiety symptoms (%)	50.38	80.00	54.80	21.30	30.29	0.000
With depressive symptoms (%)	30.23	47.50	31.00	14.90	10.91	0.004

### 3.3 Interview analysis results

The original plan for conducting student interviews had to be changed due to the update on epidemic prevention. Instead, an online questionnaire was designed with open-ended questions and a few multiple-choice questions. The questionnaire was later sent to the three class instructors who had accompanied the session after the project was completed and before the high school entrance examination. The research team utilized NVivo 12, a qualitative analysis tool, to code and analyze. A 3,600-word questionnaire answer was analyzed, and 12 open coding were formed, involving 55 references. Four main axial coding were then formed through induction and integration. Finally, the conclusions were verified using the original data verification method. The findings of the analysis are shown in Table 5 and follow.

- NMI can help relieve students’ academic-related stress. By NMI, students can adjust their emotions and relax before essential exams. Gradually, students no longer feel nervous about exams and are even able to face their failures calmly. They can actively adjust their mentality, and their academic performance has been significantly improved.
- NMI can enhance the relationship between students and teachers. Initially, reluctant students gradually became more open and willing to communicate with their peers and teachers. This led to fewer conflicts, increased harmony, and stimulated a collective consciousness among students, motivating them to work hard and improve the class. As a result, the classroom atmosphere was filled with positivity and laughter.
- Through NMI, students develop a more profound sense of self-worth and uncover their inherent strengths. Teachers were profoundly moved by the impressive advancement made by students facing learning challenges. These individuals initially struggled with lower grades and lacked the initiative to seek assistance. However, following the intervention, they gained a clearer understanding of themselves, boosted their self-esteem, and actively engaged in classroom discussions with heightened enthusiasm.
- NMI is necessary for students who can feel the positive changes in themselves and are willing to participate. It is recommended that it be continued.

**Table 5:** Coding and references of qualitative analysis.

Axial coding	Open coding	References	Example of reference
Stress	Relieve stress	10	“Activities can help students relax their nervous mood”
	Understanding stress	7	“Besides the high school entrance examination, many things are worth pursuing in life.”
	Inspires	5	“Relight their hope”
	Mental state	3	“This year of students has a more positive mental state than the previous ones obviously”
Relationship	Peers	7	“Being together brings them closer together”
	Class	4	“The class is more united and loving”
	Teacher-student	1	“Enhance teacher-student relationship”
Individual development	Emotion	7	“Get rid of some negative emotions.”
	Personality	3	“Children who used to have low self-esteem have confidence when communicating with others”
	Ability	2	“Students who usually do not perform well can take the initiative to speak and lead the team to complete activities.”
	Physics	1	“Children get exercise, too”
Academic	Academic	5	“After participating in the activity, academic performance has significantly improved.”

## 4 Discussion

Our study demonstrated a pilot empirical study focusing on the effects of adolescents with academic-related stress by real nature intervention. Research has yielded positive results. The following factors can rationalize these results.

One is the “natural” attribute of NMI, which is based on the real natural environment and interacts with real natural objects. According to the stress reduction theory, the natural environment can reduce the pressure of the experiment, and the natural environment can activate the parasympathetic nervous system through a multi-sensory experience such as vision, hearing, smell, and touch, thus reducing the level of stress hormones and relieving the stress response. At the same time, attention recovery theory can help restore our attention resources. After individuals establish a link with nature, nature stimulates non-directed attention, helps restore directed attention, reduces brain fatigue, and improves positive emotions (Beavington et al., 2021). Moreover, this kind of improvement can be achieved without extra effort. Previous studies have discovered that the positive effect of nature on our lives is not a new phenomenon, with research highlighting the restorative properties of natural exposure in both physical and psychological areas (Wang et al., 2024). Some researchers point out that the NBI structured intervention program proved to be more effective than passive immersion in nature, which was not sufficient to improve the mental status of those who were stressed or mentally unhealthy (Hunter et al., 2015; Lovell et al., 2015; Masterton et al., 2020). These findings provide additional explanations for the validity of our study. Our project design reflects four factors that make NBI effective: a natural environment, a non-demanding atmosphere, meaningful activities, and interaction with others (Johansson et al., 2022).

The second reason is to establish a mind–body connection through pre-designed and meaningful activity. That is MBI techniques, such as meditation, relaxation, etc., and some are also common techniques in NBIs, such as natural healing which improves overall health through mental conditioning. Its theoretical basis comes from the Biopsychosocial Model (Engel, 1978). The model breaks the dualism of modern medicine and health care. Whereas dualism divides the body and mind into two separate categories, the Biopsychosocial Model sees the person as a whole, emphasizing the interaction of biological, psychological, and social factors as well as human initiative. Exposure to green Spaces is associated with positive physiological effects, such as reduced heart rate and blood pressure (Twohig-Bennett and Jones, 2018); Compared with indoor exercise, outdoor exercise has a more beneficial effect on rejuvenation, engagement, tension, confusion, anger, and depression (Thompson Coon et al., 2011); Outdoor adventure activities are effective social and emotional interventions for disadvantaged youth and students (Cooley et al., 2015). Relevant review research shows that MBIs can reduce stress and increase sustained attention in middle school students, reduce depressive symptoms in adolescents, and improve mild to moderate depression (Whitfield-Gabrieli and Evins, 2023). They are commonly used to relieve anxiety and stress and are among the top 10 complementary therapies for adults and children (Black et al., 2015). In this study, although the technique incorporated was simple, students with excessive test anxiety experienced reduced negative emotions and increased concentration. Comparison of test anxiety interventions that fully used mind–body techniques (Average effect size Hedges’ *g* = 1.32, 95% CI [0.65, 1.99]; Zeng et al., 2023), such as mindfulness, Tai Chi, the effect size of this study was slightly lower (Hedges’ *g* = 1.05, 95% CI [0.58, 1.51]), but mindfulness and Tai Chi require long-term learning and professional guidance. It shows that even simple mind–body techniques can produce positive effects when combined with nature. The feasibility of the intervention was confirmed by improvements in symptomatic groups, too. This means that children and adolescents (who are not suffering from severe mental disorders) can receive necessary support through interventions easily (Sawni and Breuner, 2017).

A third reason arises from the findings of qualitative studies. The results of the interview revealed changes not shown in the quantitative data. The results found that mood, stress, relationships between students and teacher-students, and classroom atmosphere have improved. MNI was welcomed by teachers and students, as reflected by their degree of participation. These findings suggest that the intervention’s feasibility may stem from natural and mind–body characteristics and social factors based on group support. It further corroborates the Biopsychosocial Model. In other studies, good relationships have also been shown to reduce distress. For adolescents, connections with family, peers, and community have been shown to improve mental and behavioral health and critically impact development. This connection, defined by Blum et al. (2022), encompasses feelings of care, support, belonging, and closeness to others. Research by Wuthrich et al. (2020) indicates that peer and family relationships play a crucial role in reducing anxiety and stress, accounting for 21 to 22% of the variance. The findings suggest that NMI can facilitate a strong bond between students and teachers, fostering a supportive environment that alleviates academic-related stress and promotes personal development.

In addition to scientific implications, NMI also has realistic implications. This study based on the theme of rivers, aims to comprehend the local river and understand stability through change, that is, allostasis, from the ever-flowing river and the changes of the four seasons. Recognizing that change is constant and a catalyst for growth, drawing insights from nature can help students maintain a positive attitude toward their endeavors, potentially benefiting them in the long run. Drawing inspiration from natural landscapes and elements for metaphorical applications is a widely adopted strategy in natural intervention. Although there is currently a lack of systematic quantitative research to assess the effectiveness and impact of such techniques, the rich experiences accumulated in practice often lay a solid foundation for the germination and formation of new theories.

In summary, NMI provides effective, accessible, non-stigmatizing, economical help to needy students. Academically, it can relieve excessive test anxiety and promote the true expression of academic level; while helping to maintain moderate test anxiety levels, students can continue to focus on learning by improving problem-solving skills and enhancing concentration on main tasks (Lowe et al., 2008). At the same time, NMI can reduce the level of general anxiety and depression related to academic studies, thereby alleviating the negative impact of general anxiety and depression on test anxiety. In practicability, NMI allows students who may feel ashamed or unaware of the need for help to receive timely assistance. It can also avoid the impact of stigma as many teenagers may conceal their true situation due to fear of negative labels and interpersonal isolation. If students are isolated, the secondary injury may occur. NMI minimizes the negative impact of stigma. Even more, students do not have to bear additional financial burdens because of intervention.

Two limitations should be noted. Firstly, our study provides pioneering empirical evidence supporting the feasibility and acceptability of the NMI in alleviating test anxiety among adolescents. However, the study was conducted in only one school and did not include a control group. Future research could implement a cluster-randomized controlled trial (Cluster-RCT) across multiple schools to further validate these findings. Secondly, this feasibility study combines NBI techniques with MBT techniques and has initially developed a low-cost, and highly accessible intervention program. Nevertheless, further optimization and standardization are still needed in future research to ensure its use in future studies and application promotion.

This study evaluated the feasibility of NMI in the intervention of mental health issues in adolescents. The results indicate that NMI can successfully reduce excessive test anxiety and partially alleviate symptoms of anxiety and depression in middle school students. As a cost-effective and low-stigma intervention method, NMI can be easily implemented in institutions to provide mental health support to young students and other vulnerable groups. Empirical research on NMI brings enlightenment that humanity and nature are inseparable. In the process of urbanization in China or other places, urban and rural teenagers are becoming increasingly distant from the natural environment. While modern development brings convenience in life, transportation, and learning opportunities, it also brings more psychological problems due to isolation from nature. Re-establishing the connection between humans and nature, particularly the connection with the local natural place, and forming recognition, identification, and emotional inheritance through in-depth activities can not only alleviate the burden caused by modern lifestyle and academic psychological problems. Still, it can also help adolescents find motivation and sources of support for self-growth.

## Data Availability

The datasets presented in this study can be found in online repositories. This data can be found here: https://www.scidb.cn/en/s/QRRRVv.
