# Supplementary material for: Nature-based mind–body intervention for test anxiety in adolescents: a feasibility study
Source: Front Psychol. 2025 Apr 9;16:1550353. doi: 10.3389/fpsyg.2025.1550353 (PMC12015724; doi:10.3389/fpsyg.2025.1550353)
Supplement: Supplementary file 1 [file Table_1.docx]

Supplementary Material

**Research in context**

**Evidence before this study**

By searching the published literature of APA PsycARTICLES, APA PsycINFO, MEDLINE, and CNKI, we briefly review the impact of nature-based intervention (NBI) on test anxiety, anxiety, and depression in adolescents. Use English and Chinese terms such as nature-based, adolescent, anxiety, depression, and intervention through Boolean operators and parentheses to form search terms for searching. The search time is from the establishment of the database to May 2023. Inclusion criteria: 1) Quantitative or qualitative research; 2) Real natural environment; 3) Adolescents under 18 years old; 4) Chinese or English. A total of 219 English and 12 Chinese articles were obtained. After scanning the titles and abstracts, 19 articles (17 in English and 2 in Chinese) were found related to the research aim. These articles confirm that nature-based interventions can produce multiple positive outcomes for adolescents, such as children being more willing to play in greened spaces and engaging in more regular, constructive, and exploratory play behaviors, and that NBI can treat and prevent clinical depression and that potential benefits in improving mood, calming, and relaxing. Overall, NBI is a physical and mental health promotion strategy, but application research has not yet been carried out in areas related to academic stress.

**The added value of this study**

This article presents a pioneering study that explores the application of NMI in mental health issues related to academic performance among adolescents. Participants were chosen explicitly from disadvantaged communities in underdeveloped areas. The findings revealed that through a nature-based mind-body intervention, excessive test anxiety, which can hinder academic success, was reduced. Additionally, perceived stress and anxiety levels decreased, while moderate test anxiety, which can enhance academic performance, was maintained. Attaining higher education is a crucial avenue for individuals from disadvantaged backgrounds to improve their economic and social standing. However, these individuals often face barriers in accessing the necessary resources and may feel ashamed to seek help. The results of this study offer a promising, cost-effective, and low-stigma approach for this population to enhance their academic performance.

**Implications of all the available evidence**

NMI offers a low-stigma and low-cost intervention model for adolescents of all grades, races, and socioeconomic statuses. It aims to improve psychological issues related to academics, such as academic stress and mood, and to promote positive relationships between peers and teachers. NMI can be incorporated into the school's regular schedule to reduce academic workload, minimize risks associated with high-stakes exams, and promote the extensive well-being of students.
